# Core Set of Patient-Reported Outcome Measures for Measuring Quality of Life in Clinical Obesity Care

**DOI:** 10.1007/s11695-024-07381-4

**Published:** 2024-07-15

**Authors:** Phillip J. Dijkhorst, Valerie M. Monpellier, Caroline B. Terwee, Ronald S. L. Liem, Bart A. van Wagensveld, Ignace M. C. Janssen, Johan Ottosson, Bruno Halpern, Stuart W. Flint, Elisabeth F. C. van Rossum, Alend Saadi, Lisa West-Smith, Mary O’Kane, Jason C. G. Halford, Karen D. Coulman, Salman Al-Sabah, John B. Dixon, Wendy A. Brown, Ximena Ramos Salas, Sally Abbott, Alyssa J. Budin, Jennifer F. Holland, Lotte Poulsen, Richard Welbourn, Natasja Wijling, Laura Divine, Nadya Isack, Susie Birney, J. M. Bernadette Keenan, Theodore K. Kyle, Melanie Bahlke, Andrew Healing, Ian Patton, Claire E. E. de Vries

**Affiliations:** 1 Department of Surgery, OLVG Hospital & Nederlandse Obesitas Kliniek [Dutch Obesity Clinic], Jan Tooropstraat 164, 1061 AE Amsterdam, the Netherlands; 2 Department of Science, Nederlandse Obesitas Kliniek [Dutch Obesity Clinic], Huis Ter Heide, the Netherlands; 3 Department of Epidemiology and Data Science, Amsterdam University Medical Center, Location Vrije Universiteit Amsterdam, Amsterdam, the Netherlands; 4 Amsterdam Public Health Research Institute, Methodology, Amsterdam, the Netherlands; 5 Department of Surgery, Nederlandse Obesitas Kliniek [Dutch Obesity Clinic], The Hague, Gouda the Netherlands; 6 Department of Surgery, Groene Hart Ziekenhuis, Gouda, the Netherlands; 7 Department of Surgery, NMC Royal Hospital, Abu Dhabi, United Arab Emirates; 8 Department of Surgery, Nederlandse Obesitas Kliniek [Dutch Obesity Clinic], Huis Ter Heide, the Netherlands; 9 Department of Surgery, Faculty of Medicine and Health, Örebro University, Örebro, Sweden; 10 Obesity Center, 9 de Julho Hospital, São Paulo, Brazil; 11 Brazilian Association for the Study of Obesity (ABESO), São Paulo, Brazil; 12 School of Psychology, University of Leeds, Leeds, UK; 13 Scales Insights, Nexus, University of Leeds, Leeds, UK; 14 Obesity Center CGG [Healthy Weight Centre], Erasmus MC, University Medical Center Rotterdam, Rotterdam, the Netherlands; 15 Department of Internal Medicine, Division of Endocrinology, Erasmus MC, University Medical Center Rotterdam, Rotterdam, the Netherlands; 16 Department of Surgery, Neuchâtel Hospital, Neuchâtel, Switzerland; 17 Biology and Medicine Faculty, Lausanne University, Lausanne, Switzerland; 18 Department of Surgery, Department of Psychiatry and Behavioral Neuroscience, University of Cincinnati College of Medicine, Cincinnati, OH USA; 19 Department of Nutrition and Dietetics, Leeds Teaching Hospitals NHS Trust, Leeds, UK; 20 National Institute for Health Research Bristol Biomedical Research Centre, and Bristol Centre for Surgical Research, Population Health Sciences, Bristol Medical School, University of Bristol, Bristol, UK; 21 Department of Surgery, Kuwait University, Kuwait City, Kuwait; 22 Iverson Health Innovation Research Institute, Swinburne University of Technology, Melbourne, Australia; 23 Department of Surgery, Central Clinical School, Monash University, Melbourne, Australia; 24 Obesity Canada, Edmonton, AB Canada; 25 European Association for the Study of Obesity, Teddington, UK; 26 Specialist Weight Management Service, University Hospitals Coventry and Warwickshire NHS Trust, Coventry, UK; 27 Research Centre for Intelligent Healthcare, Coventry University, Coventry, UK; 28 Bariatric Surgery Registry, Central Clinical School, Monash University, Melbourne, Australia; 29 Research Unit for Plastic Surgery, University of Southern Denmark and Odense University Hospital, Odense, Denmark; 30 Lontoft, Nyhoj and Poulsen Plastic Surgery, Odense, Denmark; 31 Department of Upper Gastrointestinal and Bariatric Surgery, Musgrove Park Hospital, Taunton, UK; 32 Dutch Association for Overweight and Obesity (NVOO), Utrecht, the Netherlands; 33 People Living With Obesity Representative, Kuwait, Kuwait; 34 Patient Advocate, Trustee of the Obesity Empowerment Network, London, UK; 35 European Coalition for People Living With Obesity (ECPO), Dublin, Ireland; 36 Irish Coalition for People Living With Obesity (ICPO), Dublin, Ireland; 37 ConscienHealth, Obesity Action Coalition, Tampa, FL USA; 38 Adipositascirurgie Selbsthilfe Deutschland E.V. (Obesity Surgery Self-Help Organization), Mannheim, Germany; 39 Obesity Canada, Edmonton, Canada; 40 Department of Surgery, OLVG Hospital, Amsterdam, the Netherlands

**Keywords:** Obesity treatment, Bariatric surgery, Quality of life, Outcome reporting, Clinical practice, Patient-reported outcomes, Patient-reported outcome measures

## Abstract

**Purpose:**

The focus of measuring success in obesity treatment is shifting from weight loss to patients’ health and quality of life. The objective of this study was to select a core set of patient-reported outcomes and patient-reported outcome measures to be used in clinical obesity care.

**Materials and Methods:**

The Standardizing Quality of Life in Obesity Treatment III, face-to-face hybrid consensus meeting, including people living with obesity as well as healthcare providers, was held in Maastricht, the Netherlands, in 2022. It was preceded by two prior multinational consensus meetings and a systematic review.

**Results:**

The meeting was attended by 27 participants, representing twelve countries from five continents. The participants included healthcare providers, such as surgeons, endocrinologists, dietitians, psychologists, researchers, and people living with obesity, most of whom were involved in patient representative networks. Three patient-reported outcome measures (patient-reported outcomes) were selected: the Impact of Weight on Quality of Life-Lite (self-esteem) measure, the BODY-Q (physical function, physical symptoms, psychological function, social function, eating behavior, and body image), and the Quality of Life for Obesity Surgery questionnaire (excess skin). No patient-reported outcome measure was selected for stigma.

**Conclusion:**

A core set of patient-reported outcomes and patient-reported outcome measures for measuring quality of life in clinical obesity care is established incorporating patients’ and experts’ opinions. This set should be used as a minimum for measuring quality of life in routine clinical practice. It is essential that individual patient-reported outcome measure scores are shared with people living with obesity in order to enhance patient engagement and shared decision-making.

**Graphical Abstract:**

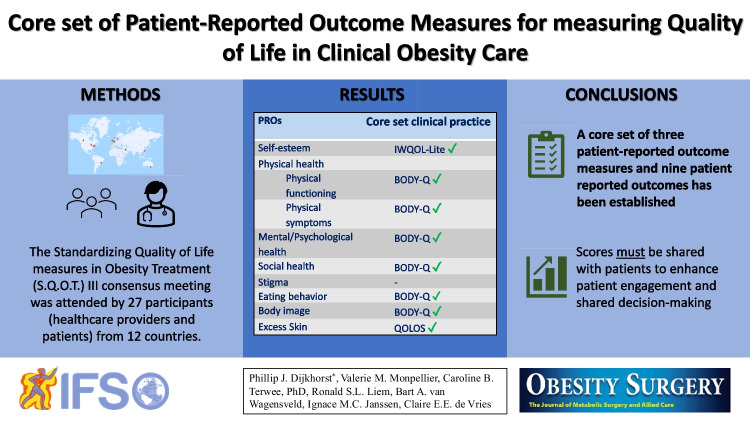

## Introduction

Obesity is chronic and impairs patients’ health and well-being [1, 2]. Treatment includes lifestyle interventions, medication, and metabolic and bariatric surgery [3]. Treatments can have a significant impact on weight and also on patients’ quality of life, ranging from body image and self-esteem to social, mental, and physical health [4–6]. Within the last decades, interest in measuring patient-reported outcomes (PROs) of surgical and non-surgical treatment has increased [7, 8], resulting in the development of numerous patient-reported outcome measures (PROMs) to assess these outcomes [9].

PROMs can be used to compare the effectiveness of interventions in research, to improve the quality of registries, and to facilitate shared decision-making in clinical practice. To date, PROMs have been used primarily in obesity research and registries [10, 11]. Nevertheless, they could also prove useful in clinical practice to assess quality of life to improve individual patient care [12, 13]. PROMs provide an in-depth understanding of patients’ experiences and of the impact of treatment on their health and sense of well-being [8]. They offer insight into patients’ symptoms and quality of life that cannot be obtained through clinical measures, such as weight, blood pressure, or laboratory tests [12, 14, 15]. Implementing PROMs in obesity treatment will enable personalized care and will improve communications between patients and healthcare providers. For example, healthcare providers could use PROMs to deliver care that aligns with patients’ priorities, and PROMs could aid shared decision-making [12].

Despite the growing interest in using PROMs in clinical obesity care, several major concerns exist. These include measuring PROs that might not be relevant for people living with obesity, the wide variation in PROMs currently available in obesity treatment, and the use of PROMs with insufficient validation evidence [6, 16–18]. To address these matters and to effectively implement PROMs in clinical practice, it is essential to develop a core set of PROs and PROMs with high-quality validation evidence [15]. Previously, multidisciplinary international consensus meetings [19], initiated by the Standardizing Quality of Life in Obesity Treatment (S.Q.O.T.) initiative, led to the development of a core set of PROs and PROMs for measuring the quality of life in research (submitted). The current study reports on the S.Q.O.T. III meeting, during which we established a definitive set of PROs and PROMs for clinical practice, and we discuss the implementation of the core set.

## Methods

The S.Q.O.T. III meeting was held on 2 and 3 May 2022, in Maastricht, the Netherlands. Six authors were involved in its organization. Participants could attend either live or online. Ethical approval was obtained from Medical Research Ethics Committees United, the Netherlands, Reference number W21.227.

The goals of the S.Q.O.T. III meeting were the following:

1. Select a PROM for the PRO for stigma.
2. Select a core set for research.
3. Select a core set for clinical practice.
4. Discuss the implementation of the core sets and their dissemination.

Selecting a PROM for stigma and the development of a core set for research were reported separately (submitted). In the current article we describe the selection of a core set for clinical practice and report on the discussions regarding its implementation.

### Participants

Every participant attending the meeting provided oral informed consent and registered on the S.Q.O.T. initiative website: https://www.sqotinitiative.com/ [20]. The participants recruited for the meeting were the following:

1. Healthcare professionals and researchers from different disciplines, experienced in obesity treatment and patient-centered outcomes research. They were recruited through international and national obesity treatment networks. Participants of previous meetings were invited again.
2. People living with obesity were involved in obesity patient representative networks and were fluent in English. They were either recruited through patient representative networks, the organizers’ networks, or through the networks of the healthcare providers. Participants of previous meetings were invited again.

### The S.Q.O.T. III Meeting

The meeting was led by an independent moderator specialized in the development of PROMs and core outcome sets (COS) who had also been involved in the S.Q.O.T. I & II meetings. The S.Q.O.T. III meeting consisted of group discussions using nominal group techniques [21], Delphi exercises [22], and anonymous voting through Voxvote (an online voting system that allows anonymous voting through computer or smartphone) [23]. These techniques, which involve systematic processing of expert opinion, can lead to substantial enhancements in both the accuracy and reliability of outcomes and are commonly employed in group meetings with the objective of attaining consensus on a particular subject. Healthcare professionals with conflicting interests were not permitted to participate in group voting. The PROs and PROMs predefined during the S.Q.O.T. I & II meetings were endorsed in the core set for clinical practice, provided the majority of participants voted for that PRO or PROM. Discrepancies in voting between healthcare providers and people living with obesity were addressed in group discussions and/or re-voting ensued. If more than 70% of people living with obesity voted in favor of or against a specific PRO or PROM, it would overrule the total number of votes. The organizers and moderators were not permitted to influence the discussions or voting rounds. They merely facilitated the meetings. The votes were described in terms of number, total percentage in favor; percentage in favor for healthcare providers, percentage in favor for people living with obesity.

## Results

### Participants

The S.Q.O.T. III meeting was attended by 27 participants: nine people living with obesity and 18 healthcare providers. The healthcare providers comprised six bariatric surgeons, two psychologists, three dietitians, two endocrinologists, three researchers, one plastic surgeon, and one physician specialized in obesity treatment. The participants represented twelve different countries from five continents. Seven participants participated online. Thirteen participants had participated in the S.Q.O.T. II meeting.

### Selection of PROs and PROMs

First, during a group discussion, the participants decided unanimously that all PROs selected for the research set were also relevant in clinical practice and should be subjected to voting. We held one voting round for each PRO domain. This resulted in PROs for self-esteem, physical function, physical symptoms, mental/psychological function, social function, eating, body image, excess skin, and stigma. Second, participants voted to select the most suitable PROM for each PRO. The PROMs selected per PRO were the Impact of Weight on Quality of Life-Lite (IWQOL-Lite, self-esteem) [24, 25], the BODY-Q (physical function, physical symptoms, psychological function, social function, eating behavior, and body image) [26, 27], and the Quality of Life for Obesity Surgery (QOLOS, excess skin [28] questionnaires (Table 1**)**. This set was identical to the core set selected for research (submitted). Below, we describe the selection process.

**Table 1:** Overview of the selection of the patient-reported outcomes and the patient-reported outcome measures for the core set

PROs	PROMs available	First selection of PROMs	Core set clinical practice
Self-esteem	IWQOL-Lite, IWQOL-Lite CT, PROS, WHO-QOL BREF	IWQOL-Lite	IWQOL-Lite
Physical health			
Physical functioning	BAROS, BODY-Q, BOSS, BQL-Index, EQ-5D-5L, GIQLI, IWQOL-Lite, IWQOL-Lite CT, M-A QOL QII, OP-scale, PBOT, PROS, QOLOS, SF-36, TRIM, WHO-QOL BREF	SF-36, IWQOL-Lite, BODY-Q	BODY-Q
Physical symptoms		BODY-Q	BODY-Q
Mental/psychological health	BAROS, BODY-Q, BQL-Index, IWQOL-Lite CT, M-A QOL QII, SF-36, TRIM, WHO-QOL BREF	BODY-Q	BODY-Q
Social health	BAROS, BODY-Q, BOSS, BQL-Index, EQ-5D-5L, GIQLI, IWQOL-Lite, IWQOL-Lite CT, M-A QOL QII, OP-scale, PBOT, PROS, QOLOS, SF-36, TRIM, WHO-QOL BREF	OP Scale, IWQOL-Lite, BODY-Q	BODY-Q
Stigma		WSSQ, SSI-B	-
Eating	BODY-Q, BOSS, M-A QOL QII		BODY-Q (eating behavior)
Body image	BODY-Q, QOLOS	BODY-Q, QOLOS	BODY-Q
Excess skin	BODY-Q, QOLOS	BODY-Q, QOLOS	QOLOS

*Abbreviations: PRO* patient-reported outcome, *PROM* patient-reported outcome measure, *S.Q.O.T.* Standardizing Quality of Life Measures in Obesity Treatment, *BAROS* Bariatric Analysis and Reporting Outcome System, *BOSS* Bariatric And Obesity-Specific Survey, *BQL Index* Bariatric Quality of Life Index, *GIQLI* Gastrointestinal Quality of Life Index, *IWQOL-Lite* Impact of Weight on Quality of Life-Lite, *IWQOL-Lite CT* Impact of Weight Quality of Life-Lite Clinical Trials, *M-A QoLQII* Moorehead-Ardelt Quality of Life Questionnaire II, *PBOT* Post Bariatric Outcome Tool, *TRIM* Treatment Related Impact Measure, *WHOQOL-BREF* World Health Organization Quality of Life Questionnaire-BREF, *QOLOS* Quality of Life for Obesity Surgery, *OP Scale* Obesity-Related Problems Scale, *SF-36* 36-Item Short Form Survey, *WSSQ* Weight Self-Stigma Questionnaire, *SSI-B* Stigmatizing Situations Inventory-brief version

#### Self-esteem

Practically all participants voted to include the PRO for self-esteem (25 votes, 92%, healthcare providers 93.8%, participants living with obesity 87.5%). They selected the IWQOL-Lite self-esteem subscale as the most suitable PROM (23 votes, 56.5%, healthcare providers 50%, participants living with obesity 100%) [24, 25]. Some healthcare providers expressed concern about the costs associated with the IWQOL-Lite questionnaire [24, 25].

We should really consider the worthiness of incorporating a PROM with associated costs into the outcome set. The self-esteem IWQOL-Lite is very similar to the BODY-Q (psychological function subscale). Therefore, it may be recommended to consider removing the self-esteem IWQOL-Lite scale due to associated costs. (Quote from an endocrinologist).

There is some overlap between the IWQOL-Lite self-esteem and BODY-Q psychological function. However, self-esteem and psychological function are clearly different, and self-esteem is a very important concept to measure in obesity treatment. Both domains should be incorporated in the outcome set. (Quote from a psychologist).

#### Physical Function

Practically all participants voted to include the PRO for physical function (25 votes, 96%, healthcare providers 100%, participants living with obesity 85.7%). They selected the BODY-Q physical function subscale as the most suitable PROM (22 votes, 90.9%, healthcare providers 100%, participants living with obesity 80%) [26, 27].

#### Physical Symptoms

Practically all participants voted to include the PRO for physical symptoms (25 votes, 96%, healthcare providers 93.3%, participants living with obesity 100%). They selected the BODY-Q physical symptoms subscale as the most suitable PROM (23 votes, 100%) [26, 27].

#### Mental/Psychological Function

Well over three-quarters of the participants voted to include the PRO for psychological function (24 votes, 87.5%, healthcare providers 86.7%, participants living with obesity 85.7%). They selected the BODY-Q psychological function subscale as the most suitable PROM (22 votes, 95.5%, healthcare providers 92.3%, participants living with obesity 100%) [26, 27].

#### Social Function

Almost three-quarters of the participants voted to include the PRO for social function (22 votes, 72.7%, healthcare providers 69.2%, participants living with obesity 85.7%). They selected the BODY-Q social function subscale as the most suitable PROM (24 votes, 70.8%, healthcare providers 61.5%, participants living with obesity 100%) [26, 27].

I like the BODY-Q (social function subscale) questionnaire because it includes situations I encounter in daily life. (Quote from a participant living with obesity).

#### Eating

Almost all participants voted to include the PRO eating (24 votes, 95.8%, healthcare providers 100%, participants living with obesity 87.5%). They selected the BODY-Q (eating behavior subscale) as the most suitable PROM (22 votes, 86.4%, healthcare providers 92.9%, participants living with obesity 66.7%) [26, 27].

I think there are a couple of important aspects to measure for eating: hunger, satiety, and how long satiety lasts. From what I hear from people living with obesity, these aspects seem to be very important for quality of life. The BODY-Q (eating behavior subscale) comes closest to measuring these aspects. (Quote from a psychologist).

#### Body Image

Two-thirds of the participants voted to include the PRO body image (24 votes, 66.7%, healthcare providers 78.6%, participants living with obesity 50%). A considerable difference was observed in the preference of healthcare providers and participants living with obesity. Several participants living with obesity mentioned that they did not want the focus of body image to be on physical aesthetics. They suggested that a PROM assessing body image should focus on the individuals’ perceptions of their own bodies. Conversely, healthcare providers reported that the PROMs available for assessing body image should accurately capture the individuals’ feelings towards their own bodies. They emphasized that body image changes drastically after weight loss treatments, and therefore, it is an important measure.

In my experience as a dietitian, body image is a very important domain because weight loss interventions have such a big effect on body image. Patients tell me that the perspective of their body changes drastically after weight loss treatments. (Quote from a dietitian).

After a group discussion, re-voting on body image resulted in more votes in favor of including body image (25 votes, 80%, healthcare providers 92.3%, participants living with obesity 62.5%), and the BODY-Q (body image subscale) was selected as the most suitable PROM (23 votes, 78.3%, healthcare providers 69.2%, participants living with obesity 100%) [26, 27].

#### Excess Skin

More than three-quarters of the participants voted to include the PRO excess skin (23 votes, 78.3%, healthcare providers 76.9%, participants living with obesity 87.5%). They selected the QOLOS (excess skin subscale) as the most suitable PROM (25 votes, 68%; healthcare providers 64.3%, participants living with obesity 71.4%) [28].

We like the QOLOS questionnaire because it covers how excess skin makes you feel, how it stops you from doing sports, and the medical consequences of excess skin such as hygiene and pain. (Quote from a participant living with obesity).

#### Stigma

Approximately three-quarters of the participants voted to include the PRO stigma (24 votes, 70.8%, healthcare providers 64.3%, participants living with obesity 87.5%). No PROM was selected because none of the available PROMs were considered suitable. Reasons included the absence of questions on general experiences of stigma, the inability to use the questionnaires longitudinally, and the absence of validation evidence in people undergoing obesity treatment. Below, one healthcare provider highlights the importance of assessing both internalized weight stigma and the effect of experiences of weight stigma.

Internalized weight stigma refers to the stigma directed towards oneself. There is a correlation between internalized weight stigma and the impact or acceptance of weight stigma. It is important to not only assess internalization, but also the effect of weight stigma, such as its impact on healthcare engagement and mental or social health. (Quote from a researcher).

This healthcare provider is supported by a participant living with obesity who also highlighted the importance of capturing the impact of weight stigmatizing experiences.

None of the questions assess the impact of stigma, they only focus on the prevalence of stigma. No scale is considered suitable for use in obesity treatment. (Quote from a participant living with obesity).

## Discussion

This study described the selection of a core set of PROs and PROMs for assessing quality of life in clinical obesity care. The process included a systematic review and a multinational consensus meeting that was attended by people living with obesity, and by healthcare providers from various disciplines. The PROs and PROMs selected were IWQOL-Lite (self-esteem) [24, 25], BODY-Q (physical function, physical symptoms, psychological function, social function, eating behavior, and body image) [26, 27], and QOLOS (excess skin) [28]. These PROMs contain good measurement properties ensuring that the measurement of quality of life provides valid and reliable outcomes [17]. This core set can be used in different cultural and geographical settings. Specifically, these PROMs are available in 19, 81, and 2 languages, respectively [24–28]. No PROM was selected to measure stigma because the available PROMs were considered unsuitable. To the best of our knowledge, this is the first multinational and multidisciplinary effort in obesity treatment to achieve the goal of standardizing the quality of life outcomes that matter most to patients. The primary objective of the core set is to improve the quality of care in obesity treatment by reflecting outcomes that are most important to patients. To this end, we incorporated the extensive input of people living with obesity throughout the development of the set. The core set represents the selection of PROMs that should minimally be used in clinical obesity care.

The same PROs and PROMs were selected for the clinical practice core set as for the research core set, which allows the same set to be used for different purposes. The PROs mostly align with the findings of Coulman et al. [29]. They identified eight themes with regard to living with the outcomes of metabolic and bariatric surgery. Despite frequent use of the SF-36 in prior obesity treatment research, it was not included in the core set as participants favored other PROMs based on face validity [16]. In a recent validation study by de Vries et al., the SF-36 was not supported by sufficient validation evidence in patients undergoing metabolic and bariatric surgery due to the irrelevance of some questions and lack of relevant items to patients among other reasons [18], aligning with the current findings. Recently, Greene et al. established a selection of PROMs to be implemented in the Metabolic and Bariatric Surgery Accreditation Quality Improvement Program in the USA [30]. They identified health, self-confidence, mobility, and everyday activities as the highest-ranking PRO domains and selected the PROMIS-10, Obesity-Related Problem Scale (OP scale), and Obesity and Weight-Loss Quality of Life Instrument (OWQOL) for inclusion. The S.Q.O.T. consensus meetings took a different approach to selecting PROMs by focusing on their suitability for the selected PRO domains. The OP scale was not considered suitable for measuring social function, and the PROMIS-10 and OWQOL were not validated for obesity treatment [17, 19]. We point out that the selection of PROMs for research purposes or clinical practice may thus far vary significantly from those used in registries. A number of healthcare providers suggested that some PROMs could be omitted from the core set and that others contained too many questions, between seven and ten, per PRO. By omitting these PROMs, feasibility might be enhanced, and less time would be needed to complete the questionnaires. According to the participants living with obesity at the meeting, omission was not necessary. They did not consider completing multiple questionnaires as burdensome, provided their purpose was explained and feedback was provided.

Using PROMs in clinical practice is increasingly becoming routine. In the treatment of cancer, for example, PROMs are used to monitor symptoms and quality of life during the preoperative and postoperative periods [31]. Their administration and interpretation led to better identification of posttreatment symptoms and problems, enhanced clinical decision-making processes, and improved communication between patients and healthcare providers [31]. The ability of PROMs to improve outcomes in cancer care was further illustrated by Basch et al. [32]. They reported that in comparison to usual care, digital symptom monitoring using PROMs significantly improves physical function, symptom control, and overall quality of life. There is even evidence that PROMs can improve survival in cancer care [33]. In obesity treatment, PROM data offer a unique opportunity to enrich consultations by redirecting the focus to those aspects that concern patients most or problems that would otherwise go undetected [34]. Sharing PROM data facilitates active patient participation in progress tracking and outcome assessment, thereby enhancing communication between patients and healthcare providers [12, 13, 35, 36]. Using PROMs in obesity treatment enables healthcare providers to deliver care that aligns with patients’ needs, and ultimately, to provide value-based and high-quality care [14, 15]. To further aid implementation of the PROMs in clinical practice, we developed a user manual for the core set (Appendix Table 2).

Despite the benefits of PROMs, there are challenges regarding their implementation. First, the participants living with obesity indicated that the lack of feedback on PROM scores was what withheld them most from routinely completing PROMs. We concluded that PROMs should only be implemented in clinical practice if the scores are shared with the patients. To facilitate this process, a digital platform is required that automatically dispatches PROMs before consultations and allows the PROM scores to be displayed on a dashboard for healthcare providers and people living with obesity to see [12]. Several electronic measurement systems are proposed in the literature, such as mobile applications, web-based systems, or email reminders [37]. Patients perceive these approaches to PROM administration as easy and user-friendly [38–40]. Second, appropriate training on PROM administration and interpretation is necessary for healthcare providers [41, 42]. The use of a minimal clinically important change (MCID), that is the smallest change in a treatment outcome that an individual would identify as important [43], could aid healthcare providers in interpreting PROM scores. The MCID from the IWQOL-Lite total score ranged from 7.7 to 12 points (depending on baseline severity) [44]. The MCID for all quality of life domains of the BODY-Q is currently being determined. Determining a MCID for the QOLOS will also be necessary.

The key strength of our study was that we involved a geographically diverse panel of healthcare providers from different disciplines, as well as participants living with obesity, most of whom were involved in patient representative networks. Initially, our goal was to involve healthcare providers and participants living with obesity in a 1:1 ratio, but on account of cancellations, we ended up with a 2:1 ratio. The moderator ensured that the participants living with obesity could voice their opinions sufficiently. There were some limitations too. First, due to the multiple subjects that needed to be addressed and the strict time schedule, prolonged discussions during the meeting were not possible. Second, some experts joined the meeting online, which may have limited their involvement during discussions. Third, one healthcare provider, involved in the development of the BODY-Q, was instructed not to vote, but she could participate in the discussions. Seeing that this restriction applied to one participant only, this is unlikely to have biased our results. Fourth, not all online participants participated in the entire two-day meeting, either as a consequence of the different time zones or because of the multiple meetings that were planned prior to the combined congress of the European Association on the Study of Obesity and the International Federation for the Surgery of Obesity and Metabolic Disorders—European Chapter.

During future meetings, we shall continue to improve the current core set of PROMs, including the development of a PROM for stigma, further validation, and additional translations of the QOLOS (excess skin) questionnaire. Moreover, the issue of costs associated with the IWQOL-Lite (self-esteem) questionnaire shall be addressed. On the contrary, the BODY-Q and QOLOS are available free of charge. Possible new PROMs shall also need to be considered in detail. We discussed these points extensively in our previous article (submitted). Future S.Q.O.T. meetings shall be necessary to determine how the PROMs should be used in registries. If this core set is adopted and implemented, it could potentially promote the collection of outcomes that are clinically important to patients and experts alike, and thus improve the quality of obesity treatment globally. Ideally, this set of PROs and PROMs should be complemented by a set of clinical outcomes that form a core outcome set for obesity treatment.

## Conclusion

A core set of PROs and PROMs for measuring quality of life in clinical obesity care was selected by a heterogenous group of participants living with obesity and healthcare providers. The set includes subscales from the BODY-Q (physical function, physical symptoms, psychological function, social function, eating behavior, and body image), IWQOL-Lite (self-esteem), and QOLOS (excess skin) questionnaires. A PROM for stigma was not selected because the available PROMs were considered unsuitable. The PROMs that were selected represent outcomes that matter most to patients and should serve as a minimum when measuring quality of life in clinical practice.

## Appendix

**Table 2:** Manual for using the core PROM set for clinical practice in obesity treatment

Name patient-reported outcome measure	Patient-reported outcome	Number of questions	Available languages	Reference publications	Request license via	Contact person additional questions
Impact of Weight on Quality of Life-Lite (IWQOL-Lite)	Self-esteem	7	81 For a list see: https://www.qualityoflifeconsulting.com/iwqol-lite-languages.html	Kolotkin, R.L. et al. (2001). Link 10.1038/oby.2001.13 Kolotkin, R.L. et al. (2002). link 10.1023/a:1015081805439 Kolotkin, R.L. et al. (2002). Link 10.1038/oby.2002.102	https://pattern.health/exchange/iwqol-impact-of-weight-on-quality-of-life/	Ronette L. Kolotkin, PhD rkolotkin@qualityoflifeconsulting.com at www.qualityoflifeconsulting.com
BODY-Q	Physical function	7	19 For a list see: https://qportfolio.org/wp-content/uploads/2023/05/BODY-Q-Translations.pdf	Klassen A.F. et al. (2016). Link 10.1097/GOX.0000000000000665 Klassen, A.F. et al. (2017). Link 10.1186/s12955-017-0802-x Dalaei, F. et al. (2022). Link 10.1111/cob.12528	Free download: https://qportfolio.org/body-q/	https://qportfolio.org/our-team/ Anne Klassen: ac.retsamcm@ssalka
BODY-Q	Physical symptoms	10				
BODY-Q	Psychological function	10				
BODY-Q	Body image	7				
BODY-Q	Social function	10				
BODY-Q	Eating behavior	9		de Vries, C.E.E. et al. (2021). Link 10.1007/s11695-021-05462-2		
Quality of Life for Obesity Surgery (QOLOS)	Excess skin	8	2 English and German	Müller, A. et al. (2018). Link 10.1007/s11695-017-2864-6	By email: mueller.astrid@mh-hannover.de	Astrid Müller: mueller.astrid@mh-hannover.de
